# Advances and Challenges in Traumatic Brain Injury from a Forensic Perspective

**DOI:** 10.2174/011570159X352125241031030110

**Published:** 2025-02-26

**Authors:** Shu-Quan Zhao, Yan-Wei Shi, Xiao-Guang Wang, Ke Liu, Hu Zhao

**Affiliations:** 1 Faculty of Forensic Medicine, Zhongshan School of Medicine, Sun Yat-sen University, Guangzhou, China;; 2 Guangdong Province Translational Forensic Medicine Engineering Technology Research Center, Guangzhou, China;; 3 Eighth Affiliated Hospital, Sun Yat-sen University, Shenzhen, China

**Keywords:** Traumatic brain injury, pathogenesis, cause of death, forensic science, cognitive impairment, alcoholism

## Abstract

Traumatic brain injury (TBI) is one of the leading causes of death and disability. Animal and clinical studies of TBI have greatly changed the clinical practice of TBI with the development and application of new technologies. However, with the development of forensic science, legal issues related to TBI continue to emerge, and it is still far from satisfactory that the practical application of relevant research findings as legal evidence in court practice. This review discusses an overview of the latest progress of TBI through neuropathological changes, secondary injury mechanisms, postmortem neuroimaging, cognitive, emotional, and behavioral impairments, biomarkers, and the effects of toxins and drugs on brain injury from a forensic perspective. Meanwhile, we highlight the interpretability and limitations of findings on TBI in legal proceedings are ongoing challenges.

## 1 INTRODUCTION

Traumatic brain injury (TBI) usually refers to the functional or structural damage of brain tissue caused by external forces, which is such a diverse and heterogeneous disease process. Even minor trauma can cause brain injury [1]. It is reported that the burden of TBI in low- and middle-income countries accounts for 80% globally, and robust trauma registries were absent in these countries. Therefore, although the incidence of TBI is estimated to be 790/100 000 annually, it is far from the real incidence [2, 3]. The incidence of TBI is rising and will become the leading cause of death and disability in the near future [4].

The causes of TBI are different in developed countries and developing countries. Falls in children under 4 years old and patients over 55 years old rank first among the causes of TBI in developed countries, while road traffic accidents are the main cause of TBI in developing countries [2, 5-7]. A large number of studies have identified risk factors for TBI, such as male, age, and alcohol. The male-dominated TBI was associated with risk-taking behavior, occupational hazards, and violence-related injuries [8]. Alcohol-related TBI is common in clinical and forensic practice, and alcohol consumption has been confirmed as a risk factor in TBI-related death.

According to the Glasgow Coma Scale (GCS) and the severity of brain injury after resuscitation, TBI was classified as mild, moderate, and severe [9, 10]. Generally, the severity of TBI is a powerful indicator of physical disability, but when it comes to mental injury, this is paradoxical because changes in cognition, attention, and other mental issues are observed even in patients with mild TBI. For example, chronic traumatic encephalopathy (CTE) is a unique neurodegenerative disease, which is closely associated with repetitive mild TBI. Patients with CTE may have various behavioral and emotional symptoms.

The development and application of neuroimaging, proteomics, RNA-sequence, and metabonomics technologies have ultimately changed the level of diagnosis, management, and therapy of TBI in clinical practice [11-22]. Indeed, TBI is very common in forensic and clinical practice. In comparison with the clinical perspective, the forensic perspective of TBI was somewhat overlooked [23]. Despite numerous TBI studies are conducted by forensic professionals, the progress of TBI in the field of forensic medicine is slow due to complex factors [24-30]. Therefore, this review aims to provide an overview of the recent progress of TBI from a forensic perspective, address the medical-legal issues of TBI, and highlight the ongoing challenges of TBI in forensic practice to attract the attention of neurologists all over the world.

## 2 PATHOPHYSIOLOGY OF TBI

Many factors can affect the clinical manifestations of TBI, the strength of the impact and the location of the hit are the triggering factors, and the sequence events such as excitotoxicity of glutamate and inflammation response play a critical role in the process of TBI [31-34]. Other factors, such as genes, also play a role in the process of TBI [33]. Most TBI cases usually recover within 10 days, but some TBI patients also suffer from long-term brain function damage, indicating the presence of chronic brain injury [35].

### 2.1 Acute Pathophysiology of TBI

The process of TBI is classified as primary and secondary injury [36]. The primary injury is associated with the physical forces and the structure of the head. The relative movement of the skull and brain is confirmed as the main mechanism in the formation of primary injury of TBI through numerous studies [37-40]. The primary injury damages the cell membranes, increases the level of K^+^ in extracellular fluid, evokes the depolarization of neurons, thereby increasing the level of glutamate neurotransmitter in the extracellular fluid. The released glutamate activates postsynaptic neurons through kainate, N-methyl-D-aspartic acid (NMDA), and A-amino-3-hydroxy-5-methyl-4-isoxazole-propionic acid (AMPA) receptors and further exacerbates potassium efflux, form an excitation loop with positive feedback. The altered ionic and increased glutamate activate protease, change the metabolism of the brain, and cause secondary injury of TBI [41].

Diffuse axonal injury (DAI) is one of the most common and important pathological features of TBI. It is defined as the extensive axonal injury of white matter after blunt violence on the head, which can lead to accelerated head movement, resulting in shear force and traction, and thus cause deep brain tissue strain injury [42]. The mechanical injury directly to the axon is called the primary axotomy, while the delayed phase of injury caused by progressive molecular and cell-linked pathological changes in the axons following initial shear stress at the time of injury is identified as the secondary axotomy, and they both contribute to the development of DAI. The latter has been considered as the key mechanism for most damaged axonal disruptions [43-45]. It is generally believed that the existence of DAI indicates the impairment of the neural circuit function within the brain [46].

The structure and integrity of cerebral blood vessels are susceptible to TBI, and the decrease in cerebral blood flow (CBF) post-TBI suggests the presence of cerebral ischemia [47]. The blood-brain barrier (BBB) may also be damaged due to the primary or secondary TBI injury [48]. Once the mechanical or pathophysiological damage occurs, inflammatory and cytokine will be produced, and microglia and glial will be activated, thereby forming a cytokine storm [49, 50]. All the events mentioned above may lead to traumatic brain edema and hypoxia, and eventually result in inevitable death.

### 2.2 Chronic Pathophysiology of TBI

The crucial role of neuroinflammation and oxidative stress have been carefully addressed in the acute phase of TBI, while their role in the chronic phase of TBI was still confusing [51]. The increased level of Tumor Necrosis Factor-α (TNF-α) released by activated astrocytes was confirmed as the initiator of inflammatory responses, and the activated inflammatory responses in turn increased the level of TNF-α [52]. Although the increased level of TNF-α plays a crucial role in the normal electrical circuit of the brain, the higher level of TNF-α post-TBI has been proven to impair synaptic function, thereby inducing hyperexcitability and epileptic episodes [53]. However, the activation of astrocytes, microglia, and glial is common during the recovery phase following DAI, and the underlying mechanism of epilepsy post-TBI still needs further study to clarify [51].

The accumulation of β amyloid (Aβ) and tau protein in the brain is the common pathological change between TBI and neurodegenerative diseases. Although TBI has been proven to be a risk factor in the process of neurodegenerative diseases, including Parkinson's and Alzheimer's, the underlying mechanism is still a paradox [54-56]. The crucial role of oxidative stress and neuroinflammation was identified through animal experiments and the Cohort studies of human patients [57-59]. Oxidative stress is the main reason for the development of Alzheimer's disease (AD), antioxidant therapy in animals with TBI can reduce the occurrence of neurodegenerative processes [60]. The persistence of activated microglia is common in TBI patients and animals even in years following the initial injury, this inflammation may accelerate the process of neurodegenerative disease [58]. The inflammation cytokines in TBI may increase the concentration of β-site amyloid precursor protein cleaving enzyme 1 (BACE1) and thus lead to the accumulation of Aβ. Furthermore, the persistent inflammation response might affect the function of microglia and neurons [61].

However, most studies diagnose Alzheimer's disease based on clinical manifestations rather than autopsy or biomarkers. To date, the increased Aβ and tau observed in animal and human autopsy studies has somewhat built a link between TBI and neurodegenerative diseases, and the level of Aβ and tau will decrease normally once TBI recovers [34]. The underlying mechanisms in TBI-induced neurodegenerative diseases are still confusing and need further studies to clarify [50].

## 3 LONG-TERM OUTCOME OF TBI

### 3.1 Post-traumatic Stress Disorder (PTSD)

PTSD is a mental disorder caused by exposure to extreme traumatic events, affecting approximately 8% of the population and causing a heavy economic burden on individuals and society [62]. It can be caused by a single traumatic event or repeated traumatic events, characterized by invasive memories, avoidance of trauma-related stimuli, repeated nightmares, and persistent hyperarousal [63]. PTSD and TBI have some similar neuropsychiatric symptoms, including anxiety, irritability, personality changes, and memory problems, which make differential diagnosis a challenge [64]. Previous studies indicated TBI was a key risk factor for PTSD, and PTSD also played an important role in mental injury post-TBI. Indeed, the comorbidity of PTSD with TBI is common in clinical and forensic practice [65-67]. Generally, PTSD has a negative dose-response relationship with TBI, and it is prone to occur in Moderate TBI (mTBI) cases rather than severe TBI [68-70].

Emotion regulation is recognized as a complex process by which individuals experience and express emotions. There is significant overlap in clinical manifestations and neurological consequences between PTSD and TBI, most of which can be categorized as emotion regulation disorders [71]. Dysfunctional emotion regulation in TBI and PTSD is caused by the affected emotion regulation circuit [72]. Animal and human imaging studies have confirmed that the amygdala, prefrontal cortex (PFC), and hippocampus are the main brain regions involved in PTSD [73-75]. Sub-cortical structures and PFC constitute a core circuit for emotion regulation, and molecular changes induced by physical and/or emotional trauma can directly affect this circuit, leading to the common emotional dysregulation characteristic of PTSD and TBI. The underlying mechanisms can be summarized as inflammatory/excitotoxic, degeneration, cell death, and limbic circuit malfunctions [76-78].

PTSD post-TBI itself is a diagnostic challenge of considerable concern in forensic psychiatry [79]. The ease with which subjective symptoms of PTSD can be exaggerated and altered, and the fact that psychiatric symptoms and conditions are self-reported, greatly increases the difficulty of correctly diagnosing PTSD. Although recent studies have shown that genes also play a role in the process of PTSD after TBI, the progress in revealing the neurobiology of PTSD has been limited due to the inability to obtain and analyze the brain tissue of PTSD patients [62]. So far, brain banks of PTSD patients have been established, which allow high-throughput transcriptomic and proteomic studies of specific brain regions associated with PTSD. Single nucleotide polymorphisms (SNPs) and epigenetic changes could be identified by Genome-Wide Association Studies (GWAS), which will accelerate the further elucidation of the genetic basis and molecular pathways of PTSD in the future.

### 3.2 Cognitive Dysfunction

Cognitive dysfunction is a common long-term adverse neuropsychological outcome in patients with TBI [80]. Many cognitive deficits have been reported in patients with mTBI, including executive function, learning and memory, attention, and processing speed [81]. A single concussion can disrupt the neurological system behind cognition and is easily detected in the early post-injury period, but long-term outcomes are unclear due to a lack of research [82]. Other neuropsychological symptoms have been reported to recover within two weeks, whereas cognitive deficits may persist for three months or more after injury [83]. Approximately 15% of TBI patients have long-term cognitive deficits even one year later, and more than half of patients with a single mTBI display a variety of measurable cognitive impairments one year after the initial injury [82]. Even 10 years after TBI, patients may have cognitive impairments, which are strongly associated with post-TBI quality of life [84-86].

TBI has been proven to be a risk factor for hypopituitarism, and partial hypopituitarism is a consequence of mTBI [87]. Symptoms in patients with chronic brain injury and hypopituitarism overlap with each other, and include memory deficits, decreased scores on neuropsychological tests, and worsening of mental symptoms [88]. It is well known that almost all types of brain injury cause hormonal disturbances leading to endocrine and metabolic dysfunction [89].

The hypothalamic-pituitary axis plays an important role in neuroendocrine function, and the arcuate nucleus (ARC) is a peripheral regulatory center for glucose and lipid metabolism [90, 91]. Patients with TBI-induced hypopituitarism exhibited various patterns of deficits in the neuropsychological tests [88]. A link between growth hormone deficiency (GHD) and cognitive impairment was also identified in numerous animal and human studies [92-95]. The early onset of GHD impairs learning and memory, which can be prevented by supplementing GH in animals and patients [95, 96]. However, assessment of cognitive performance in GHD patients and controls show little difference, which has led to confusion about the link [97]. In general, GHD frequently occurs in patients with TBI, and administering GH treatment can increase the levels of neurotransmitters in the cerebrospinal fluid (CSF) and improve the health and cognitive function of patients [98].

Metabolic disorders are abnormal metabolic processes including glucose, liquids, and acid-base imbalances. Diabetes mellitus, diabetic ketoacidosis and hyperlipidemia confirm the association between cognitive dysfunction and metabolic disorders [99]. It is widely recognized that metabolic changes occur in the central nervous system (CNS) after TBI, which may play a critical role in cognitive dysfunction post-TBI. Common underlying mechanisms of cognitive impairment induced by TBI and metabolic diseases have been identified as BBB leakage, insulin resistance, and inflammation response [99-101]. The association between inflammatory response and metabolic regulation is important for the stability of the internal environment. Imbalance in the internal environment causes metabolic disorders, which can lead to an aggravated inflammatory response. Since insulin is a central factor in immune and inflammatory signaling pathways, a large number of studies have focused on insulin resistance and inflammatory responses [101]. Therefore, the metabolism-related inflammatory response has become a new research field in the mechanism of cognitive dysfunction induced by TBI.

To date, the potential association between TBI and cognitive dysfunction is still paradoxical. Although the history of TBI and the existence of cognitive dysfunction could be confirmed by various objective examinations, there are still many puzzling questions about the direct link between them in the field of forensic science.

### 3.3 Chronic Traumatic Encephalopathy (CTE)

CTE is a unique neuro-degenerative disorder closely associated with repetitive mild TBI [102]. Although it first appeared in the medical community 100 years ago as “punch drunk” syndrome, it has only recently been accepted by the public [103]. Generally, it has been derived primarily from the clinicopathological evaluation of American football players with behavioral or emotional symptoms. To date, it has been defined as sports-related TBI, which can occur in various sports, including boxing, professional wrestling, ice hockey, and rugby [103-106]. Repeated head injuries have been reported to play a key role in the process of CTE, with age of exposure younger than 12 years and cumulative injury dose confirmed as risk factors for the development of CTE [103].

Immuno-excitotoxicity is considered one of the main mechanisms of CTE. Glutamate and inflammation cytokine released post-first mild TBI may contribute to a hyper-responsive state of microglia. Once activated, over-activated microglia would release more inflammation cytokines and excitotoxins [107]. Repeated mTBI might maintain the pro-inflammatory state of microglial, form persistent immuno-excitotoxic activity, and damage the function of neurons.

In general, CTE is characterized by a wide variety of clinical presentations, which may lead to misdiagnosis even by experienced clinicians, and postmortem pathology is the gold standard for diagnosing CTE [103]. CTE can be diagnosed by histopathology, including the characteristics of phosphorylated tau accumulation in neurons located in peri-vascular areas in deeper cortical layers, subpial and superficial regions, with or without phosphorylated tau accumulation in astrocytes [102]. The middle frontal gyrus, inferior parietal lobule, superior rtemporal gyrus, and middle temporal gyrus are the highest positive areas for CTE screening and should be routinely sampled at autopsy [108].

Although CTE has been extensively studied recently through animal models and human autopsy brain tissue, the evidence is insufficient to support it as a unique disease entity [109]. With the exception of athletes in related sports, CTE is quite rare in the general population [102]. However, an association between CTE and repeated mTBI has been well established. The legal issue in this topic is how to evaluate the medical intervention in sports events, and who has the right to allow the player with mTBI to return to the arena [108].

Generally, CTE is a rare event in forensic practice, because accidents and cardiovascular disease account for most of the sports-related death [110]. The diagnosis of CTE in forensic practice without conventional observation of the key brain regions is also a challenge for neuropathologists. However, it is reported that there are about 3.8 million sports-related concussions occurred in the USA and more than 36% of professional footballers suffered multiple episodes of concussion. It is expected that shortly, the diagnosis of CTE may increase greatly and come into the forensic field [111].

## 4 THE CAUSE OF DEATH IN TBI

TBI is known to be a risk factor for mortality, even increasing mortality 10 years after the first injury [112]. However, most studies have ignored the severity of TBI [112-114]. In 2022, Byers *et al*. conducted a propensity-matched cohort study on 426,580 adult veterans including 213,290 TBI patients, and concluded that TBI increased the mortality of patients in the first six months post-injury, and the TBI severity had an impact on the cause and time of death [115]. At the same time, most of the studies have focused on the relationship between TBI and mortality, while few studies focused on the relationship between specific causes of death and TBI [113-116]. The role of cortisol and brain-derived neurotrophic factor (BDNF) in the CSF may be its underlying mechanism. Therefore, increased CSF BDNF and cortisol, and decreased serum BDNF were strong predictors of the mortality of TBI patients in the short term [117-119].

Previous studies have also confirmed the link between suicide and traumatic brain injury [120-122]. Patients with moderate TBI often commit suicide by drug overdose in the first 6 months post-initial injury [115, 120]. Although the underlying mechanisms of suicide are far from being clarified, the risk factors were identified as emotion, well-being, and distress-oriented factors [123]. Individuals have been reported to attempt suicide once they experience a setback, and a diagnosis of traumatic brain injury may have an impact on their cognition, leading them to attempt suicide [123]. However, the association between suicide and TBI is far from being confirmed by forensic practice, let alone used as court evidence.

In recent years, with the development of medical technology, the concept and criteria of brain death have been adopted and revised. Brain death is defined as the complete and permanent loss of brain function by Neurologic Criteria in 2020 [124]. Generally, this is a clinical diagnosis, which is far from death criteria in forensic practice. In forensic practice, the cause of death should answer what content and which region of the brain could account for the underlying death mechanism. Epidural hematoma and subdural hematoma are also common clinical manifestations of TBI and may lead to death. Forensic pathologists should carefully analyze these situations to determine the exact cause of death [125]. The contusion in the brainstem or herniation that compresses the brainstem is the common cause of death in forensic practice. However, this may be the direct cause of death in TBI in the short term, and the association between the cause of death in the long term post-TBI and TBI was hard to establish in forensic practice.

## 5 FORENSIC EXAMINATION OF TBI

### 5.1 Postmortem Imaging

The rate of forensic and clinical autopsy is declining worldwide. It is reported that only about 12% of the fetuses and children have been autopsied, which has put the autopsy rate at a historical low [126, 127]. The reasons were attributed to the unwillingness of direct relatives, moral or religious restraint, and concerns about the destruction of the corpse due to autopsies [128]. Due to the rapid development of radiology technology, virtual autopsy technology (postmortem noninvasive imaging) has been successfully applied in forensic practice and proved to be a useful tool for the diagnosis of the cause of death [129]. A large number of studies have shown that virtual autopsy can provide information similar to the forensic autopsy, and is more acceptable than traditional autopsy [130-132]. Here, we carefully review the relevant studies and summarize the applications, advantages, and limitations of these technologies in Table **1** [129-148].

Bone X-rays provide information not only about bone structure and integrity but also about bone development and health [133]. It is a mandatory screening program for children under 2 years of age. All children under 3 years old or older should perform a three-dimensional reconstructed Computed Tomography (CT) of the head according to the needs of the case investigation [134]. With the development of CT and its application in forensic practice, multiplane CT, especially three-dimensional reconstruction of multiplane CT, is more accurate than skull X-ray in the diagnosis of skull fractures. At this time, the X-ray examination should be abandoned [135-148]. So far, postmortem computed tomography (PMCT) has become a well-established complement to forensic practice, which is routinely performed before autopsy [138]. The advantages of PMCT have been illustrated in many studies because it can detect major injuries corresponding to autopsy, and thus reveal the real causes of death [140-142].

In general, PMCT demonstrates a higher sensitivity than autopsy [132]. Moreover, the analysis of skull fracture morphology in PMCT can provide clear information about force intensity and crime scene reconstruction. The image data acquired by PMCT can be stored indefinitely and can provide accurate information on skull fractures. In the future, we need to further study the skull injury caused by blunt force through finite element method.

PMCT has a significant advantage in the determination of bone fracture injury, while its diagnostic value was limited in soft tissue and brain injury. Although the application of postmortem computed tomography angiography (PMCTA) has addressed these issues, it is still a challenge from a forensic perspective [143-145]. Due to the higher sensitivity of MRI in the diagnosis of parenchymal and soft tissue, postmortem magnetic resonance imaging (PMMR) has been introduced into forensic practice and has been proven to be a useful auxiliary tool for autopsy [146-148]. Compared with PMCT, PMMR not only provides better detection of brain injury but also maintains a clear image of the brain *in situ* structures, thus providing more information than traditional autopsy [149].

Many studies have compared the diagnostic accuracy of postmortem imaging technologies with that of traditional autopsy and found that various imaging technologies have different application fields and values [132-138]. The selected postmortem imaging should be based on the requirement of the case. For example, PMCT is used to identify bone fracture, PMCTA for soft tissue, and PMMR for the brain. Previous studies have also recommended the use of PMCT+PMMR or PMCTA to detect vascular lesions [130]. In addition, the widespread use of PMCT has changed the choice and method of autopsy. Clinical pathology interest has shifted from multi-organ biopsies to PMCT with minimally invasive autopsies, which have the same diagnostic value as traditional autopsy in determining the cause of death in the adult autopsy [150].

Although postmortem imaging has been widely used in forensic practice, considering the complicated factors in the diagnosis of the cause of death, it is unwise to make the decision solely based on the imaging results. Many studies have also emphasized the disadvantages of postmortem imaging [143-148]. Firstly, the edema and exudation of the brain induced by the freeze-thaw of the corpses can increase the volume or herniation of the brain, which is very common in forensic practice [151]. Secondly, although the decomposition of the brain can play a role in the identification of postmortem death interval, it complicates the diagnostic value of postmortem imaging [139]. Thirdly, the absence of secondary signs after trauma and differences in imaging parameters are the characteristics of postmortem imaging different from clinical radiology. Knowledge and experience in clinical radiology may not be suitable for postmortem imaging. In a word, postmortem imaging is a novel field intersecting forensic pathology and radiology. Whether forensic pathologists receive radiology education or radiologists receive forensic pathology education, which is better and who can make the diagnosis, remains a challenge for the development and application of postmortem imaging [141].

### 5.2 Biomarkers

With the rapid development of TBI diagnostic technology and the further understanding of the underlying mechanism of TBI, biomarkers in TBI are also emerging. From a clinical perspective, biomarkers play an important role in the diagnosis, monitoring the evolution, and predicting the outcome of TBI [152]. Generally, the serum is the most commonly used sample in clinical practice. Numerous studies have confirmed the potential value of protein biomarkers in TBI, including neuronal, axonal and astroglial biomarkers [153-157]. However, up to date, only S100 calcium-binding protein B (S100B) has been included in clinical guidelines, and the limited use of S100B in TBI in clinical practice was also stressed [154]. Overall, S100B was useful in selecting patients who should undergo CT examination, but its potential value in predicting the prognosis of TBI was paradoxical [155].

In the past decade, postmortem biochemical analysis has developed rapidly and proved to be a useful tool in the diagnosis of death caused by hypothermia, myocardial infarction, and anaphylactic shock in forensic practice [156]. Based on the studies focused on the biomarkers in TBI in clinical practice, numerous forensic organizations seek selected biomarkers, including glial fibrillary acidic protein (GFAP), tau, and S100B, to evaluate their potential role in the diagnosis of TBI death (Table **2**) [157-171]. In addition, postmortem biochemical analysis of biomarkers in TBI can provide information on survival time and postmortem interval (PMI) time [162]. Samples used for postmortem biochemical analysis were usually collected from cadavers with early postmortem changes, and almost all human tissues could be used as detection samples, but whether the biochemical analysis results were affected by the age, sex, hemolysis, perinatal rescue, and storage conditions were still unknown [163]. To date, many studies have used serum, CSF, and paraffin-sectioned brain tissues to evaluate the potential value of selected TBI biomarkers in clinical practice, but progress has been limited [164-166].

From a clinical perspective, the serum is the most commonly used sample in the biochemical analysis of TBI. However, in forensic practice, only a few studies focused on the serum biomarkers of TBI due to the presence of postmortem hemolysis. The potential value of GFAP, S100B, BDNF, Interleukin 6 (IL 6), C-reactive protein (CRP), and microtubule-associated protein tau (MAPT) as serum biomarkers for TBI has been reportedly investigated. Although the increased levels of GFAP, S100B, and neuron-specific enolase (NSE) in serum have been confirmed in TBI cases, other studies showed the increased level of GFAP may be attributed to the prolonged near-death period, and the increased level of most serum biomarkers were also detected in other cause of death cases [157-176]. Indeed, the serum was not an ideal sample for biochemical analysis of TBI biomarkers in forensic practice.

Due to the anatomical connection between CSF and the brain, and the easy availability of CSF, CSF is an ideal sample for detecting the biomarkers of TBI, even if the autopsy result is negative [177, 178]. CSF has been proven to be a useful sample for the diagnosis of TBI in forensic practice because brain protein can enter CSF directly or through the lymphatic system. To date, some biomarkers in CSF, including GFAP, NSE, S100B, Neurofilament light chain (NFL), myelin basic protein (MBP), and BDNF, have been identified in the diagnosis of TBI in forensic practice, while most of the samples were collected from corpses within 48 hours [161-178]. The timeline of changes in the levels of these biomarkers can provide information about the interval time between injury and death.

It is generally accepted that the value of CSF in forensic practice is more limited with the increased time of death. If brain tissue is well preserved, immunohistochemistry (IHC) could play a role in validating of biochemical results and provide additional information for forensic neuropathological interpretation of TBI [164-178]. To date, although numerous studies have found the limited potential value of selected proteins as biomarkers in the diagnosis of TBI in forensic practice because the expression level of these biomarkers changed with the interval time between injury and death, it could provide information about the interval time. For example, β-app IHC is a standard protocol for the diagnosis of DAI and was considered a potential biomarker in the diagnosis of TBI in forensic practice. However, even the value of β-app as a diagnostic biomarker of DAI has also been in doubt as it may be secondary to ischemia and hypoxia, and the characteristics of β-app IHC in DAI, ischemia, and hypoxia were so confusing that even experienced neuropathologist could make mistakes in distinguish them [179].

Although IHC has displayed advantages in biomarkers of identifying TBI in forensic practice, the limitations were also stressed in numerous studies. First, the brain is large and consists of many functional regions. Considering that sampling the whole brain is expensive and time-consuming, the selected structure regions in TBI cases are limited [179]. Second, the IHC results were unstable and unreliable using the samples collected in routine forensic practice. Although some authors recommended that using an improved fixed method of brain tissue, such as fixation in 70% alcohol after 24 hours in formalin, could improve the results of IHC in TBI, there is still a lack of consensus on the sampling and fixation methods of brain tissue [29]. Finally, the expression level of selected biomarkers in the brain may vary depending on age, sex, disease, and other factors [180].

With the development of technology, corrosion casting, micro-computed tomographic imaging, serial block face-scanning electron microscopy, and multiplex immunohistochemical analysis combined with AI were introduced to investigate the underlying mechanisms in the process of various diseases, and throw light on the future directions of medicine development [181-184]. However, most of the studies were performed on clinical samples rather than forensic autopsy samples. Due to the numerous factors that affect the results in forensic practice, the application of these techniques still needs further validation.

In addition, the development and application of RNA sequences is also a complementary method in routine forensic practice [185]. The small size and considerable stability of RNA, even in disaggregated samples, suggests that it may be a potential biomarker in forensic practice [186, 187]. Generally, microRNA is single-stranded fragments with 18 to 24 nucleotides, it is quite stable even in extreme conditions, such as cold, pH, and chemical disposal, and it is unaffected by RNase due to the role of RNA-binding proteins in lipoprotein complexes and extracellular vesicles. Previous studies have shown that it was a useful tool for the detection of PMI, trauma, toxic abuse, and drowning in forensic practice [187-189].

It has been reported that about 70% of microRNAs are expressed in CNS and play a role in the function of the brain [21]. In general, the special micro-RNA in the brain can enter the circulation through BBB by macrovesicles, exosomes, and lipoproteins. Reduced miR 21, miR 92, and miR 16 have been observed in serum and paraffin brain tissue section of TBI cases and showed their value as biomarkers of TBI in forensic practice [170]. Numerous clinical studies investigated the potential value of various micro-RNAs as TBI biomarkers, whereas, only a forensic study involved 26 TBI decedents and 45 controls. And it revealed that hsa-miR-124-3p, hsa-miR-138-5p, and hsa-miR144-3p were increased in the TBI group compared to the control, and the expression level of these miRNAs showed consistency in three brain regions (coup area, contrecoup area, and the corpus callosum) [190-193]. Indeed, micro RNAs have shown their advantages in exploring the underlying mechanisms of TBI in forensic practice, but they also have their limitations. For example, the results obtained cannot be interpreted because collection protocols and analytical methods vary from one laboratory to another. Moreover, the characteristics of the sampled corpse may affect the expression of microRNA [185]. In conclusion, the application of biomarkers in TBI in forensic practice still faces many unsolved problems and needs efforts all over the world.

## 6 EFFECT OF TOXINS AND DRUGS ON TBI

TBI and substance abuse are common in forensic practice, and the association between TBI and substance abuse has been confirmed to some extent in previous studies [194-196]. Alcohol tops the list of reported drugs of abuse, accounting for approximately half of patients with TBI [197]. the majority of alcohol-abusing TBI patients are involved in traffic accidents. Positive detection of alcohol and higher blood concentrations of alcohol in TBI patients have been confirmed to be associated with lower cognitive function and a higher incidence of disability [198-200]. For every 1.2% reduction in alcohol consumption, the incidence of severe TBI is reduced by 4.3% in males and 2.4% in females [201].

**Fig. (1):**
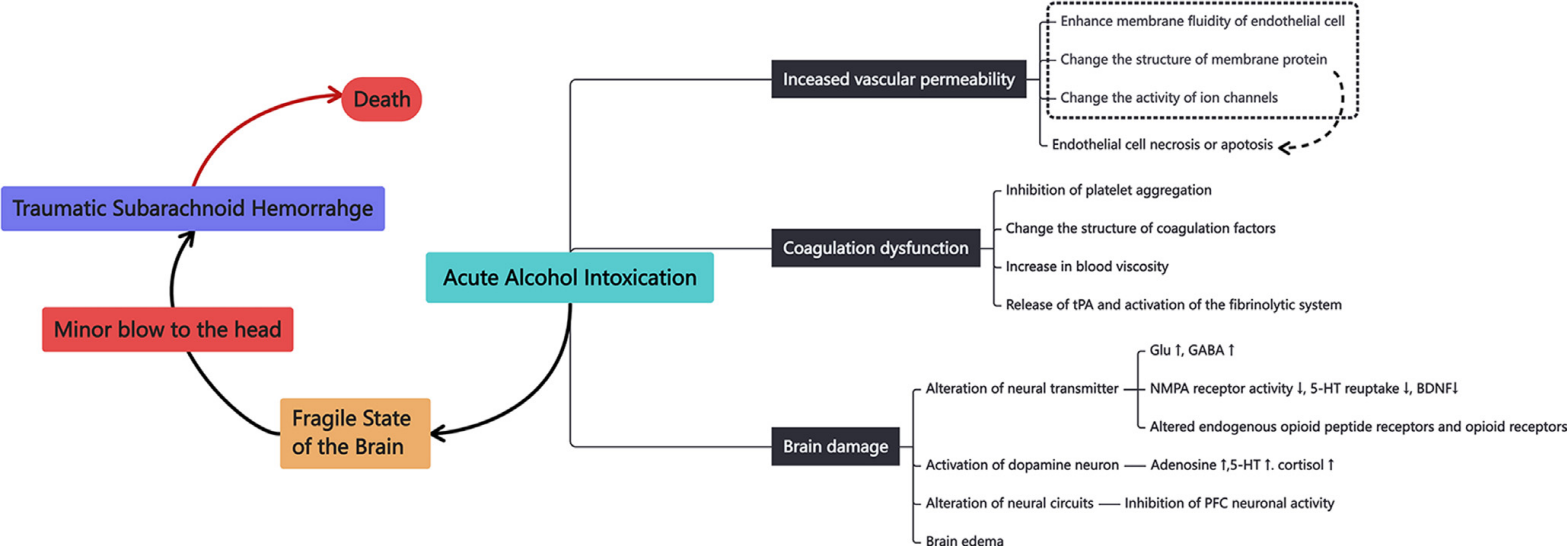
The mechanism of immediate death after a slight blow to the head after acute alcoholism. Acute alcoholism can cause coagulation dysfunction, increase vascular permeability, and damage brain neurons, ultimately leading to a fragile state of the brain. Once lightly hit, TSAH may occur, resulting in irreversible death.

Alcohol-related TBI is also common in forensic practice, and alcohol overdose may be one of the risk factors for TBI-related death [202]. Occasionally healthy young people are intoxicated die immediately after a minor blow to the head or a fall. Disseminated traumatic sub-arachnoid hemorrhage was found by autopsy. In this case, how to evaluate the role of external forces and alcohol in causing death is a challenge. It is reported that chronic alcohol abuse could damage the cerebral vascular wall, leading to vascular thickening and sclerosis, thus making it fragile to external force [203]. However, the underlying death mechanisms do not illustrate as brain contusion and skull fracture were absent in such cases [204]. The underlying death mechanism may be attributed to the over-activation of the coagulation system, increased permeability of brain vessels, and brain damage which formed a fragile brain state aroused from the acute alcoholism (Fig. **1**) [204].

Drugs for treating TBI may also have a role in forensic practice. TBI alters the pharmacokinetics of the drugs used in TBI, so that more doses are needed to achieve the therapeutic level [205-207]. Warfarin is one of the most commonly anticoagulant used drugs for the treatment of TBI, but it has been associated with an increased risk of fatal outcomes after a simple fall [208-210]. Anticoagulation of patients can increase the incidence of fatal intracranial hemorrhage by 10 times even for minor head injuries [209]. Associated adverse complications have been reported in more than 80% of patients treated with warfarin in combination with prescribed medications, and paracetamol-containing products were the most involved drugs in this condition [211-215]. The underlying mechanisms remain to be elucidated from both clinical and forensic perspectives.

The legal aspects of traumatic brain injury are complicated by toxins and drugs detected during the injury. Toxins and drugs seldom were the major cause of death at this time, and it may interrupt the injury and the subsequent outcomes. However, the association between the toxins, injuries, and their subsequent outcomes remains so confusing that no one can figure it out even from a medical point of view. From a forensic perspective, the post-mortem detection and distribution of toxins and drugs may pose a major challenge in determining the true nature of such toxicity or drugs in TBI cases.

In the past decade, pharmacogenetics and pharmacogenomics have attracted the attention of forensic pathologists, particularly in sudden death related to therapeutic doses of clinical drugs [216-218]. Previous studies have confirmed that genetic polymorphisms of CYP2C19, CYP2D6, and CYP3A4 alter drug metabolism and transport, leading to unexplained death in such cases [216]. It is suggested that the combination of pharmacogenetics and pharmacogenomics can help forensic pathologists identify the true cause of death and avoid making mistakes.

## 7 FUTURE CHALLENGES

TBI is a major public health problem with forensic significance. Interpreting TBI findings in legal proceedings requires effective communication between forensic experts and legal professionals. Presenting complex scientific information to a non-specialized audience and addressing the limitations of forensic reports in court are ongoing challenges. The complex legal issues related to TBI, especially the accurate assessment of brain injury and disability, mental injury induced by TBI, and the relationship between injury and disease or between drugs or toxins and injury, are full of great challenges.

Firstly, effectively distinguishing between TBI and non-traumatic brain injury, such as natural diseases or pre-existing conditions, is itself a major challenge. Forensic experts must carefully evaluate the circumstances, medical history, and autopsy findings to establish the causal relationship between trauma and brain injury. Secondly, in clinical practice, patients with TBI often exaggerate their condition or feign illness to obtain more compensation. Developing effective and objective identification techniques for exaggeration or malingering is a matter of top priority for forensic medicine. Thirdly, drugs or toxins detected at the time of injury, and the postmortem changes of these cases all complicated the circumstances of the legal issues of TBI, which still need further studies. Finally, although we have a better understanding of the pathogenesis of TBI, the exact underlying mechanism of mental injury induced by TBI is still unclear. The expertise of mental injury is often controversial and questioned in court, which also needs to be confirmed by further research.

Regarding the issue at the forensic scene, it is a complex task to differentiate between accidental and non-accidental causes of TBI, especially in cases of suspected child abuse or intimate partner violence. Forensic experts must rely on multidisciplinary approaches, including medical evaluations, imaging screening, and thorough scene investigations, to determine the mechanism and intent of the injury. Additionally, estimating the timing of TBI is crucial in forensic investigations, especially in cases involving intentional injury leading to death. However, accurately determining the exact time of injury remains challenging, because the signs and symptoms of TBI may gradually develop or delay, and antemortem injury and postmortem injury may coexist and have different injury characteristics.

## CONCLUSION

TBI is one of the leading causes of death and disability. Due to our further understanding of the pathogenesis of TBI, the level of clinical diagnosis, treatment, and rehabilitation has improved significantly. However, legal issues related to TBI continue to emerge, and the application of relevant research findings as legal evidence in court is still far from satisfactory. Therefore, more reliable and objective techniques need to be developed to assess the severity of brain injury and disability, determine the impact of natural diseases, toxins, and drugs on brain injury, and effectively diagnose exaggeration and malingering. Here, we provide an overview of the recent progress of TBI from a forensic perspective and highlight the interpretability and limitations of findings on TBI in legal proceedings.

## Figures and Tables

**Table 1:** Postmortem imaging in TBI.

**Technology**	**Application**	**Strength**	**Limitation**	**Suggestion**	**References**
X ray	Bone fracture	Information of bone structur, development, biometry and abnormalities	Difficult to assess on fracture of the ribs	Skull X ray in all children younger than 2 years, skull X ray is useful	[120, 121]
PMCT	Bone fracture, post-mortem interval, personal identification	“Non-destructive” documentation, identify foreign bodies and anatomical positions, digital archives for FEA	Soft tissues injuries, cardiac causes of death, image artifacts	PMCT with 3 D reconstructions of the skull in children younger than 1 year and in older children	[116-129]
PMCTA	Soft tissue and organ findings	Vascular events and haemorrhages, cause of death	Postmortem changes, iatrogenic artifacts	-	[130-132]
PMMR	Soft tissue and organ findings	Cardiac related death, DAI, cerebral infarction, cerebral contusion, and brainstem injury with mTBI, provide an overview of brain tissue anatomy *in situ*	Long inspection time, the high costs of the equipment and maintenance, the complexities of the technology	-	[133-135]

**Table 2:** Forensic biomarkers of TBI.

**Sample**	**Biomarker**	**Numbers of Cases**	**Survival Time**	**Interval Time**	**Method**	**Results**	**References**
Serum, CSF, Cortex	NSE and S100B	92	19d	5~148 h	ECLIA, IHC	Increased	[161]
Urine and saliva	MAPT	27	0	24 h	Elisa	Increased	[162]
Corpus callosum, parasagittal white matter, brainstem, and the hypothalamus	Orexin-A	49	Immediate death and 1.5 months	Between 12 and 24 h	IHC	Decreased	[163]
Frontal cortex, hippocampus and cerebellum	IL-6 and GFAP	75	0~145d	Within 6d	IHC	Increased	[160]
CSF	CK and CK-MB	92	-	-	Elisa	Increased	[166]
CSF	BDNF, GFAP, IL-6, NGAL, NSE and S100B	100	Within 2 h	-	ECLIA	GFAP, IL-6 increased	[165]
Contusion areas	NFL	25	0~30 d	Within 24 h	IHC	Increased	[29]
Brain	HMGB1 and RAGE	29	6-72 h	-	IHC	Decreased HMGB1, increased RAGE	[167]
Serum	GFAP	129	Within several hours	110 ± 67 h	ELISA	Increased	[156]
CSF, frontal lobes	GFAP,NF和MBP	38	-	12-24 h	ELISA, IHC	Increased	[157]
brain	CD34	90	-	7 d	IHC	Increased	[168]
Cingulate cortex and corpus callosum	Dynein, dynactin, and kinesin	38	-	within ~24-48 hours	IHC	Increased	[164]
Contusion areas	AQP4, CD68, IBA-1, HIF-1α, GFAP, CD15	145	0~30 d	-	IHC	Increased	[169]
Serum, CSF	GFAP, BDNF, NGAL	84	0~72 h	148h	Elisa	Increased	[158]
Contusional cerebral cortex region	SGLT1, SGLT2	19	0~121 d	0~7 d	Wb	Increased	[170]
Serum	S100B, GFAP, NSE, BDNF, IL-6, CRP, PCT, ferritin, sTNFR1, LDH	20	-	0~2 d	ECLIA	Increased	[159]

## LIST OF ABBREVIATIONS

AD: Alzheimer's Disease
ARC: Arcuate Nucleus
BBB: Blood-brain Barrier
BDNF: Brain-derived Neurotrophic Factor
CBF: Cerebral Blood Flow
CNS: Central Nervous System
CRP: C-reactive Protein
CSF: Cerebrospinal Fluid
CT: Computed Tomography
CTE: Chronic Traumatic Encephalopathy
DAI: Diffuse Axonal Injury
GCS: Glasgow Coma Scale
GHD: Growth Hormone Deficiency
IL 6: Interleukin 6
MBP: Myelin Basic Protein
NFL: Neurofilament Light Chain
NSE: Neuron-specific Enolase
PFC: Prefrontal Cortex
PMI: Postmortem Interval
PMMR: Postmortem Magnetic Resonance Imaging
PTSD: Post-traumatic Stress Disorder
SNPs: Single Nucleotide Polymorphisms
TBI: Traumatic Brain Injury
TNF-α: Tumor Necrosis Factor-α
